# “Smart Emergency Call Point” Enhancing Emergency Medical Services on University Campuses

**DOI:** 10.1017/S1049023X23006647

**Published:** 2024-02

**Authors:** Korakot Apiratwarakul, Lap Woon Cheung, Chatkhane Pearkao, Dhanu Gaysonsiri, Kamonwon Ienghong

**Affiliations:** 1. Department of Emergency Medicine, Faculty of Medicine, Khon Kaen University, Khon Kaen, Thailand; 2. Accident & Emergency Department, Princess Margaret Hospital, Kowloon, Hong Kong; 3. Department of Emergency Medicine, Li Ka Shing Faculty of Medicine, The University of Hong Kong, Pokfulam, Hong Kong; 4. Department of Adult Nursing, Faculty of Nursing, Khon Kaen University, Khon Kaen, Thailand; 5. Department of Pharmacology, Faculty of Medicine, Khon Kaen University, Khon Kaen, Thailand

**Keywords:** Emergency Medical Services, personal communication, response time, technology, universities

## Abstract

**Introduction:**

The “Smart Emergency Call Point” is a device designed for requesting assistance and facilitating rapid responses to emergencies. The functionality of smart emergency call points has evolved to include features as real-time photo transmission and communication capabilities for both staff and emergency personnel. These devices are being used to request Emergency Medical Services (EMS) on university campuses. Despite these developments, there has been a lack of previous studies demonstrating significant advantages of integrating smart emergency call points into EMS systems.

**Study Objective:**

The primary goal of this study was to compare the response times of EMS between traditional phone calls and the utilization of smart emergency call points located on university campuses. Additionally, the study aimed to provide insights into the characteristics of smart emergency call points as a secondary objective.

**Methods:**

This retrospective database analysis made use of information acquired from Thailand’s EMS at Srinagarind Hospital. The data were gathered over a period of four years, specifically from January 2019 through January 2022. The study included two groups: the first group used the phone number 1669 to request EMS assistance, while the second group utilized the smart emergency call point. The primary focus was on the response times. Additionally, the study documented the characteristics of the smart emergency call points that were used in the study.

**Results:**

Among the 184 EMS operations included in this study, 60.9% (N = 56) involved females in the smart emergency call point group. Notably, the smart emergency call point group showed a higher frequency of operations between the hours of 6:00am and 6:00pm when compared to the 1669 call group (P = .020). In dispatch triage, the majority of emergency call points were categorized as non-urgent, in contrast to the phone group for 1669 which were primarily cases categorized as urgent (P = .010). The average response time for the smart emergency call point group was significantly shorter, at 6.01 minutes, compared to the phone number 1669 group, which had an average response time of 9.14 minutes (P <.001).

**Conclusion:**

In the context of calling for EMS on a university campus, the smart emergency call points demonstrate a significantly faster response time than phone number 1669 in Thailand. Furthermore, the system also offers the capability to request emergency assistance.

## Introduction

The Emergency Medical Services (EMS) process begins when there is a medical emergency incident reported to request assistance. Dispatchers and call takers will conduct a preliminary medical history to assess the severity of the illness and then dispatch the nearest and most suitable EMS operating units to provide care.^1,2^ The most commonly used communication channel for this is typically the telephone, with specific numbers varying from one country to another.^3–5^ In Thailand, the telephone number used for receiving reports of medical incidents is 1669, which operates around the clock, covering all areas of Thailand.^6^ It’s worth noting that previous studies^7,8^ have demonstrated that achieving shorter response times positively impacts the evaluation of symptoms, diagnosis, and treatment procedures, especially in cases of time-sensitive medical conditions.

A university campus is a sprawling community with a substantial population comprising both staff and students. There is a high volume of vehicles around the university area. Therefore, managing the area is an administrative challenge to achieve maximum efficiency and safety.^9^ The emergency situation is an unexpected event that can affect a person or a community with many people. Most of the emergencies found in university areas are health, safety, information, and criminal activities.

Khon Kaen University (KKU; Khon Kaen, Thailand) serves as a prominent example of a sizable academic institution. Established as a major university in the northeastern region of Thailand in 1964, KKU spans a total area of 8.8 square kilometers. Within its grounds, the university accommodates a substantial population, with 37,122 students and 11,445 staff members. Managing the diverse needs and potential emergencies within such a dynamic and extensive environment is a multifaceted task.

A “smart emergency call point” is a device used to seek assistance during emergency situations. In the United States, smart emergency call points are located on some urban campus. The emergency hotspot is commonly called a “blue light phone” which can set as well as university campus goals.^10^ These smart emergency call points provide several benefits such as providing a place to call for help and respond to incidents promptly. At present, the capability of intelligent smart emergency call points has been developed to be able to talk between staff and emergency responders as well as to broadcast images. Real-time audio enables staff to respond appropriately and efficiently to emergencies. The adoption of this technology is a new innovation in Thailand universities. Therefore, studying the benefits of smart emergency call points, especially in EMS, will serve as an important model for the use of technology for rapid response in other areas.

The aim of this study was to compare response times between requests made through smart emergency call points and telephone calls on a university campus. Moreover, the study assessed how people used the smart emergency call points on the university campus.

## Methods

### Design and Setting

This retrospective database analysis was conducted at Srinagarind Hospital in Khon Kaen, Thailand, and it involved various EMS operations. Srinagarind Hospital serves as the region’s medical school and handles approximately 2,500 EMS operations annually. Within the EMS unit, there is a dispatch center responsible for managing and coordinating two motorcycle ambulances and six van ambulances.

The study was conducted within the campus of KKU, which is a government university located in the northeastern region of Thailand. The KKU campus encompasses various educational and residential sectors. For EMS services on the campus, there is a single agency responsible for coordination and response.

### Participants

Data collection for this study spanned from January 2019 through January 2022. All information pertaining to patients transported by EMS throughout the study period was obtained using both a smart emergency call point and a phone in the KKU campus area. Cases with incomplete or insignificant data, as well as those involving situations where EMS operations extended beyond the campus area without recognizing a patient, were excluded from the analysis.

### Data Collection

Demographic information, including age and gender, as well as operational data such as activation time and response time, were collected for all patients. Additionally, data related to the patient’s type (trauma, non-trauma) and the type of the first procedure performed on scene were recorded using a national standard operation record form for Thailand EMS and were recorded in the EMS database of Srinagarind Hospital. To ensure data accuracy, these records were evaluated by two independent and experienced emergency physicians. Following this initial assessment, a double data entry process was conducted. In cases where there were discrepancies in the data, a senior emergency physician reviewed and resolved the discrepancies to ensure data consistency and accuracy.

### Definition

Activation time was used to describe the interval between dispatch and the departing of the ambulance. Response times were measured from the moment the call was received by Thailand’s 1669-center to when the EMS responder arrived on the scene.

In this investigation, one single synchronized clock was located in the dispatch center to record and standardize time intervals. The dispatcher used telemedicine tools to monitor EMS activities in real time. Each operation’s time data were entered into the EMS database through computer systems.

### Sample Size

The sample size for this study was determined based on previous study findings.^8^ The authors concluded that a minimum of 92 samples would be required to conduct a statistically valid analysis. The statistical analysis was conducted using IBM SPSS for Windows version 27.0, a software product developed by IBM Corp. (Armonk, New York USA) and licensed by KKU for this study. Categorical data were presented in the form of frequencies and percentages, providing an overview of the distribution of categorical variables within the sample. For continuously distributed data, means and confidence interval were employed to summarize central tendencies and the degree of data dispersion. To assess the associations between categorical variables, the chi-square test was applied. Statistical significance was determined based on a two-tailed P value, with a threshold of significance set at less than 0.05. This approach is commonly used to evaluate whether observed associations between categorical variables are statistically significant.

### Smart Emergency Call Point

**Figure 1.**
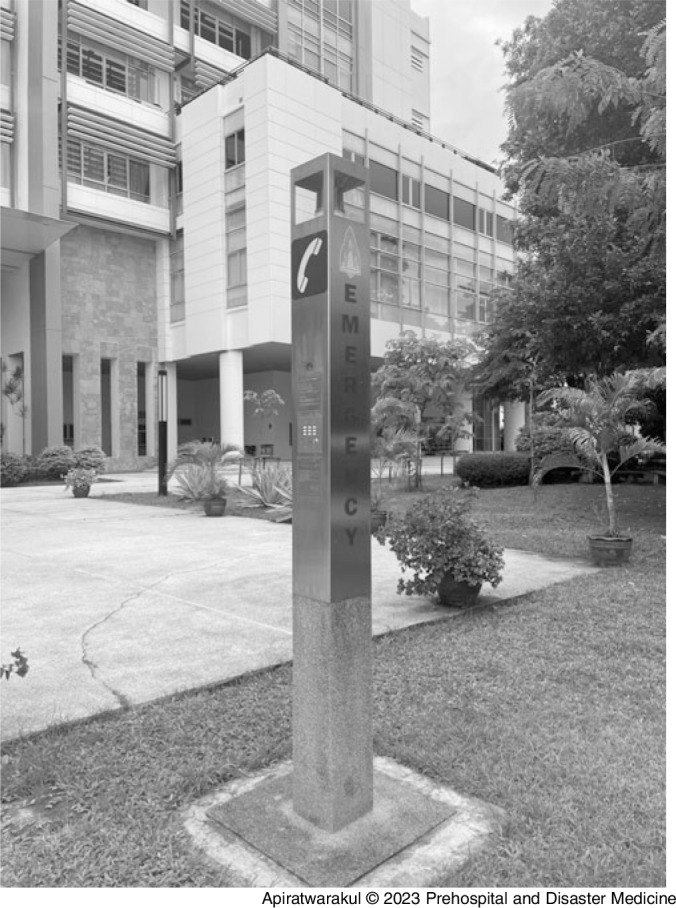
Smart Emergency Call Point.

In the KKU area, twelve smart emergency call points have been installed since 2017 with the ability to communicate between the person reporting the incident and the security center 24 hours a day. Regarding usage, individuals in need of assistance can simply press a single button to request help. Upon activation, there will be a flashing light signal on the top of the smart emergency call point. The person reporting the incident can directly contact the security command center, both visually and audibly, with real-time communication. In addition, the smart emergency call points are equipped to show their location for easy identification and quick access (Figure 1).

### Ethical Considerations

The Khon Kaen University Ethics Committee for Human Research (HE651366) issued ethical approval for this study. Because patient confidentiality had been ensured and patients were identified only by their unique study number rather than their names, the need for informed consent from the patients was waived.

## Results

In this study, a total of 184 EMS operations from the hospital’s database were analyzed over the course of a four-year period. The characteristics of which are shown in Table 1. A total of 60.9% (N = 56) were female in the smart emergency call point group. Operation times were most frequent in the daytime (6:00am to 6:00pm) in the smart emergency call point group, which differed from the phone number 1669 group (P = .020). In the smart emergency call point group, trauma patients accounted for 67.4% of all cases in the study. The majority of emergency call points were non-urgency level in dispatch triage, as opposed to the phone number 1669 group which primarily involved urgent cases (P = .010).

**Table 1.** Characteristics of the Subjects (N = 184)

Feature	Category	Smart Emergency Call Point N = 92	1669 Phone Number N = 92	P Value
Gender, N (%)	Female	56 (60.9)	54 (58.7)	.882
Age (years)	Mean (SD)	20.3 (1.2)	20.4 (1.6)	.622
Operation Time, N (%)				.020^a^
	6:00am to 6:00pm	64 (69.6)	41 (44.6)	
	6:00pm to 6:00am	28 (30.4)	51 (55.4)	
Type of Patients, N (%)				.520
	Trauma	62 (67.4)	58 (63.0)	
	Non trauma	30 (32.6)	34 (37.0)	
Year of Student, N (%)				.684
	1	20 (21.7)	18 (19.6)	
	2	28 (30.4)	29 (31.5)	
	3	17 (18.5)	18 (19.6)	
	4	7 (7.6)	5 (5.4)	
	Other Included Non-Student	20 (21.8)	22 (23.9)	
Level of Dispatch Triage				.010^a^
	Resuscitation	5 (5.4)	12 (13.0)	
	Urgency	42 (45.7)	65 (70.7)	
	Non-Urgency	45 (48.9)	15 (16.3)	

a Statistical significance.

The activation time, which represents the interval between dispatch and the departure of the ambulance, was found to be 1.45 minutes for the smart emergency call point group and 1.40 minutes for the phone number 1669 group, as shown in Table 2. Importantly, the average response time in the smart emergency call point group was significantly shorter, standing at 6.01 minutes, compared to the phone number 1669 group, where the average response time was notably longer at 9.14 minutes (P <.001). This suggests that using smart emergency call points on the university campus led to faster response times for EMS compared to traditional phone calls.

**Table 2.** Comparing Operation Times between Smart Emergency Call Points and the 1669 Phone Number to Request Emergency Medical Services (N = 184)

Time	Smart Emergency Call Point (min) N = 92		1669 Phone Number (min) N = 92		P Value
	Mean	95% CI	Mean	95% CI	
Activation Time	1.45	(1.20–1.62)	1.40	(1.15–1.70)	.752
Response Time	6.01	(4.25–8.01)	9.14	(8.40–13.20)	<.001^a^

a Statistical significance.

In Table 3, the analysis of how people used the smart emergency call points on the university campus revealed that the majority accounted for trauma traffic accidents (48.8%), health-related with non-trauma (23.6%), public utility (15.0%), and finally, requests for information (7.9%).

**Table 3.** Characteristics of Smart Emergency Call Point Use (N = 127)

Situation Used	Frequency	Percent
Trauma Traffic Accident	62	48.8
Health-Related with Non-Trauma	30	23.6
Public Utility	19	15.0
Information	10	7.9
Criminal	4	3.1
Fire	2	1.6

## Discussion

This study provides a valuable comparison of response times for EMS using two different reporting channels: the conventional telephone number 1669 and the innovative smart emergency call points. It highlights the unique challenges faced by university campuses with varying design elements, roads, meeting places, and housing that differ based on the surrounding socioeconomic conditions. Additionally, university campuses have complex building layouts that make it difficult to access patients. Having a channel to let EMS know the patient’s location was a crucial tool for getting to patients quickly in cities with many different building types.

The study found that the informant’s average age was younger than that obtained in prior studies.^11,12^ The obvious reason is that while other studies in the field of EMS were open to all applicants and the sample group would primarily be made up of a group of seniors, this study solely focuses on EMS in the university setting, leading to a majority of the study’s participants as students.

The study’s findings regarding the operation times of smart emergency call points and phone numbers align with the practicality of these communication channels. Smart emergency call points, prominently located in front of buildings and along roadsides, are more likely to be used during the day when they are easily visible to the general public. On the other hand, phone numbers are the more common choice for reporting emergencies at night when individuals typically reside indoors and have access to a phone. These insights highlight the importance of considering the convenience and accessibility of reporting mechanisms in different contexts and times of day to optimize emergency response systems.^13–15^

The majority of patients who are reported through smart emergency call points are non-urgent level patients in terms of triage. Patients have to be able to physically walk in order to reach and report an occurrence at a smart emergency call point. In contrast, being informed via the 1669 emergency number could often indicate more severe symptoms. Instead of walking to report an incident at a smart emergency call point, most individuals select to use rapid and convenient access to EMS via a telephone number.

The study’s findings are quite significant. They indicate that using a smart emergency call point for incident notification leads to faster EMS response times compared to calling 1669, primarily due to the smart emergency call point’s unique coordinates. Additionally, closed-circuit television cameras (CCTV), which can improve visibility, are included with the smart emergency call point. The patient’s symptoms can be more accurately evaluated by the dispatch center. This suggests that smart emergency call points can revolutionize the way emergency responses are handled by providing critical visual information to responders in real-time.

Regarding the positive effects of smart emergency call points, it was found that they are commonly used by people who need to report an emergency incident on health-related matters due to both accidents and medical conditions. Additionally, smart emergency call points have advantages when used in a variety of contexts, covering utility emergencies, information requests, criminal investigations, and fire alerts.^16–18^ Furthermore, smart emergency call points contribute significantly to the spatial development of complex university building structures by facilitating the swift arrival of EMS, security personnel, and disaster response units during emergencies. Their integration with CCTV enhances the visibility and surveillance capabilities of the immediate area, thereby bolstering campus safety and fostering a culture of confidence among both students and campus staff.^19^ In essence, smart emergency call points play a multifaceted role in enhancing emergency response, security, and overall safety on university campuses.

The adoption of this technology by a wide range of educational institutions throughout the world is helping to cultivate a sustainable smart city culture, which prioritizes the rapid reporting of incidents and the prompt response to emergencies. These smart emergency call points promote a sustainable smart city culture and serve as pivotal tools in enhancing safety, security, and emergency management within educational campuses, ultimately contributing to the creation of safer and more efficient learning environments.

## Limitations

This study has several limitations worth noting. Firstly, it was conducted retrospectively, meaning that some data were unavailable or incomplete. Additionally, the study only covers one university area, so the findings might not be easily generalized to different campuses with various building structures and student populations. The study also specifically examined how one EMS responded in a variety of ways when providing services inside the context of the public health system. Lastly, the effective utilization of smart emergency call points relies on having a responsible unit available to receive notifications and respond 24 hours a day, which may not always be feasible in all settings.

## Conclusions

The utilization of smart emergency call points on the university campus has demonstrated a significantly faster response time than phone number 1669, which illustrated the positive effects in EMS access. Furthermore, the system also offers the capability to request both medical and non-medical emergency assistance. This integration of technology and emergency services is seen as advancement for addressing the challenges of urban development, including structures and city planning for sustainable development.
